# Structural connectivity in a paediatric case of anarchic hand syndrome

**DOI:** 10.1186/s12883-015-0477-z

**Published:** 2015-11-14

**Authors:** Anna P. Basu, Peter Neal Taylor, Eva Lowther, Elizabeth O. Forsyth, Andrew M. Blamire, Rob J. Forsyth

**Affiliations:** Institute of Neuroscience, Newcastle University, Newcastle upon Tyne, NE1 7RU UK; School of Computing Science, Newcastle University, Newcastle upon Tyne, NE1 7RU UK; Institute of Cellular Medicine and Newcastle Magnetic Resonance Centre, Campus for Ageing and Vitality, Newcastle University, Newcastle upon Tyne, NE4 5PL UK; Magdalene College, University of Cambridge, Cambridge, CB3 0AG UK; Institute of Neuroscience, Newcastle University, Sir James Spence Institute, Royal Victoria Infirmary, Newcastle upon Tyne, NE1 4LP UK

**Keywords:** Anarchic hand, Corpus callosum, Tractography

## Abstract

**Background:**

“Anarchic hand” is a rare condition characterised by non-volitional, goal-directed movements of one arm. We report a case with analysis of structural and functional connectivity.

**Case presentation:**

A 15 year old girl developed intermittent symptoms of intermanual conflict or anarchic hand as a result of traumatic brain injury during which she sustained a callosal bleed. Resting-state fMRI and DTI tractography were performed at a stage when symptoms had largely resolved.

**Conclusion:**

Structural connectivity between homologous superior frontal areas and functional connectivity between homologous posterior cingulate areas were significantly reduced, which may have contributed to causation. Tractography demonstrated new indirect connections between supplementary motor areas via the cerebellum, which we propose contributed to symptom resolution.

## Background

“Anarchic hand” is a rare condition characterised by non-volitional, goal-directed movements of one arm and is extremely rare in childhood. We report a case of paroxysmal anarchic hand episodes following traumatically-acquired callosal injury at the age of fifteen, with analysis of functional and structural connectivity. Although qualitative tractographic studies of callosal disconnection syndromes have been published [1, 2] ours is the first to attempt quantitative assessment of connectivity changes.

## Case presentation

A fifteen-year old right handed girl sustained a severe closed head injury as a pedestrian in a road traffic accident. CT head showed a large haematoma above the corpus callosum. Her left arm was immobilised in a plaster cast for four weeks for fracture management. She was discharged after 5 weeks, having apparently recovered well. Past medical history was remarkable for a seizure disorder with temporal lobe semiology that had presented two years previously due to a histologically confirmed ganglioma, which was completely resected.

Shortly after the plaster cast was removed she found herself momentarily having difficulty letting go of the telephone from her left hand after a call. One week later she picked up a ham in a supermarket with both hands but was obliged to buy it as her left hand would not release it. When attempting to open a door with her right hand, she would find her left hand simultaneously shutting it. When playing dominoes, her left hand picked out a different domino than she had intended to play and placed it on the table. Once when putting up a poster, her left hand pulled the poster off the wall. At worst, events occurred three times a week. No arm elevation, self-injurious or purposeless movements were noticed and there was no leg involvement. She would manage the episodes by moving away from the situation producing the conflict. She reported occasional subjective premonitory awareness a few seconds prior to an attack and would try to take preventative action such as putting her hand in her pocket. There was no overt denial of ownership of her left hand at any point: rather her description of events was that her left hand “seemed to have a mind of its own”. Event frequency diminished with time and ceased by twenty months.

Examination findings 18 months after the accident were unremarkable apart from a mild qualitative change in pinprick sensation in her left hand and forearm in a glove distribution to just above the elbow. Somaesthetic transfer and cross-replication of hand postures were performed normally. Praxis to verbal instructions was normal when using the left hand, as was performance of alternating motor sequences.

Her uni- and bimanual dexterity scores on an adapted 9-hole pegboard test were within normal limits for age. An EEG performed at five months showed excess theta and delta activity in left temporal leads without seizure activity; no anarchic hand events were captured.

### Imaging and analysis

The opportunity afforded by the need for follow-up imaging of the resection bed of her previous ganglioglioma was taken to perform additional resting-state functional MRI and diffusion tensor imaging (DTI) sequences twenty months after injury. Scans were collected using a Siemens Verio 3T scanner with an 8-channel head coil. A resting-state fMRI sequence was acquired using a gradient-echo EPI sequence with 34 slices and 128 repetitions over 4.25 min. DTI was performed using a standard spin-echo EPI DTI sequence with 64 diffusion directions (b-factor 1000s.mm^−1^ and 2 b0 volumes). The in-plane resolution was 2 mm, the slice thickness was 2.2 mm.

Voxelwise diffusion was computed using DTK. The brain was parcellated into 83 regions and deterministic tractography was applied [3] to create structural connectivity matrices (quantified by number of connecting streamlines) using standard tools from the Connectome Mapping Toolkit Pipeline [4]. Fibre tracts with extreme angles (>60°) and implausible lengths (>50 cm) were excluded. SMA regions were defined by alignment with the AAL atlas [5] using DSI-Studio [6]. All alignments were confirmed by visual inspection.

Findings were compared to five age-matched controls from the public-domain NKI database [7]. We tested for statistical significance only considering connections present in all controls. Mahalanobis distances for the patient from the controls were adjusted for multiple comparisons using false discovery rate (FDR) correction [8]. In view of the patient’s previous tumour surgery we also disregarded all connections involving nodes in the left temporal lobe in both the patient and controls.

Analysis of resting state fMRI data was performed using the MELODIC tool (www.fmrib.ox.ac.uk/fsl). We focussed on the component identified as the sensory-motor network in the literature [9]. Functional connectivity was inferred by pairwise examination of the time-domain correlation of the resting-state fMRI BOLD signal between nodes, with results expressed as Mahalanobis distances from the five NKI controls.

### Findings

**Fig. 1:**
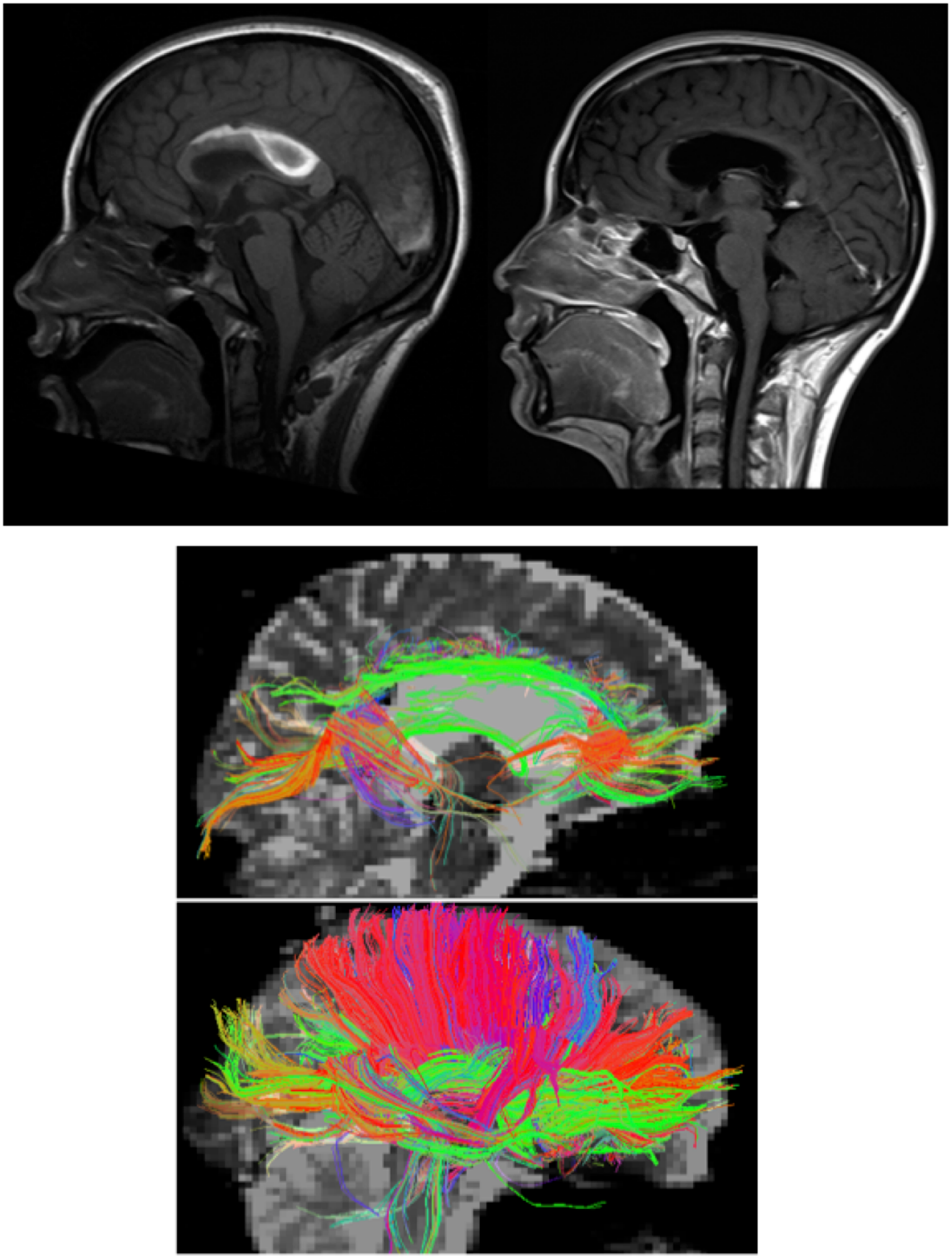
*Top*. Sagittal T1 MRI brain images of patient. Left: day 17, showing a large callosal haematoma. Bilateral subdural haematomas were also present as were post-surgical changes related to the previous ganglioglioma resection with ex-vacuo dilatation of the left temporal lobe. However there was no evidence of diffuse axonal injury and no evidence of cortical damage. Right: 12 months later, showing resolution of the haemorrhagic changes but marked atrophy of the body of the corpus callosum. MRI brain at 20 months showed no further changes (not shown). *Bottom*. Deterministic tracking of the callosal tract based on two regions of interest defining the corpus callosum on sagittal planes to the left and right of midline. The patient is above with a control below for comparison. The majority of interhemispheric direct connections (red) are lost except at the extremes of the genu and splenium

Follow up MR imaging at 12 and 20 months post injury showed marked callosal atrophy (Fig. 1 top). DTI assessment of the callosal tracts at 20 months suggested loss of the majority of the direct interhemispheric connections, with some preservation at the extremes of the genu and splenium (Fig. 1 bottom).

Surprisingly, despite the loss of the main callosal fibres, only one change in structural connectivity met the significance criterion. Connectivity between homologous superior frontal areas was significantly reduced in the patient vs controls (Mahalanobis distance −3.57; *p* < 10^−4^). A number of increased connection strengths within the left temporal lobe were presumed to reflect consequences of her previous surgery. Time-domain correlation of the resting state fMRI signal showed a marked reduction in functional correlation between the homologous posterior cingulate areas (Mahalanobis distance −14.2; *p* <10^−4^).

**Fig. 2:**
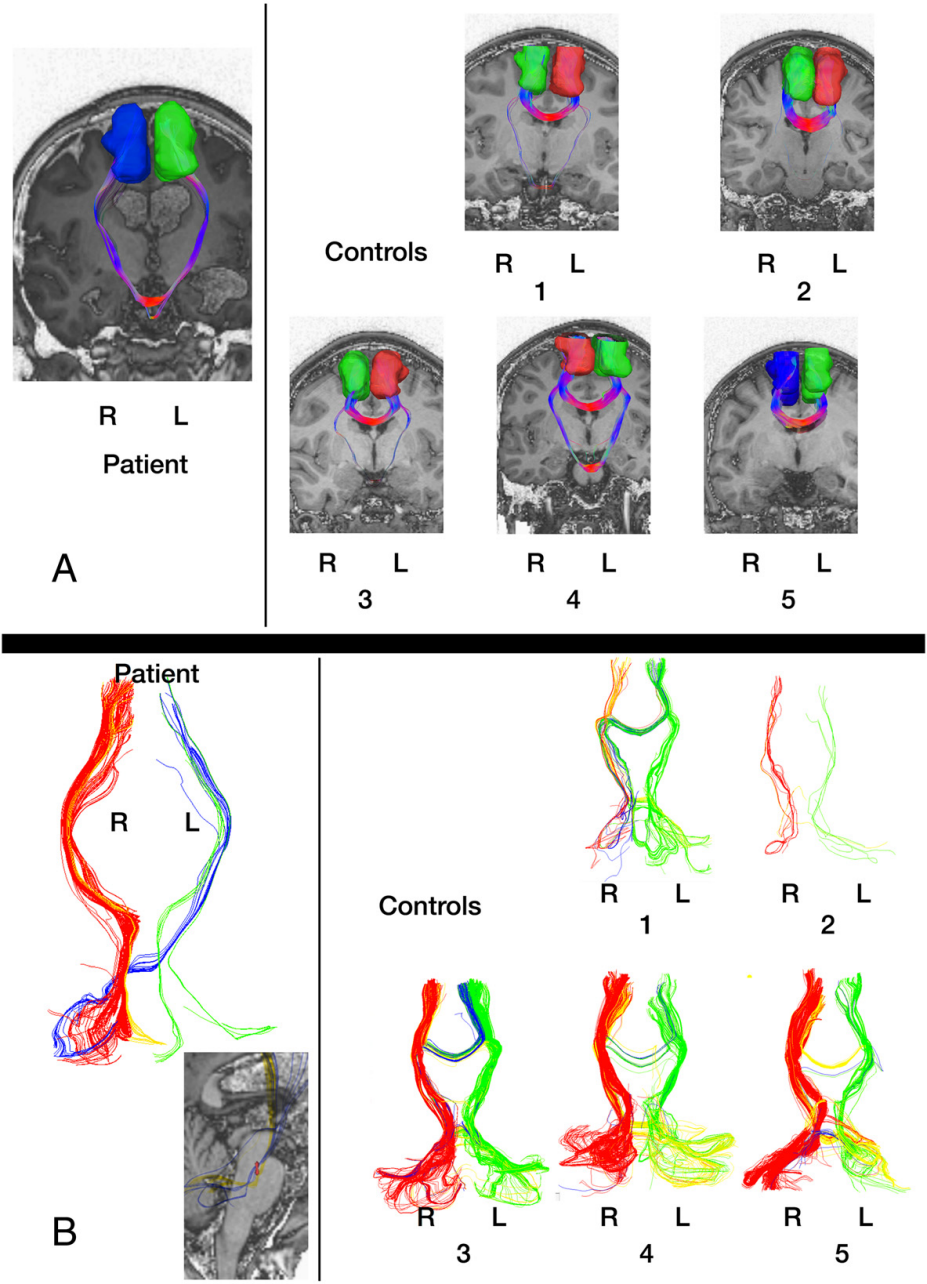
**a** (*top*) Tractography findings in patient (*left*) and five age-matched controls from the NKI dataset [7] (*right*) with seeds placed in Supplementary Motor Areas **b** (*bottom*) Tractography in patient (*left*) and the five controls (*right*) between cerebellar hemispheres and SMA. Uncrossed connections are shown in green (*left*) and red (*right*); crossed connections are shown in yellow (*left cerebellum to right SMA*) and blue (*right cerebellum to left SMA*). The T1 image inset bottom left shows (*small red bar*) the level at which the “blue” pathway crosses the midline in the sagittal plane

**Fig. 3:**
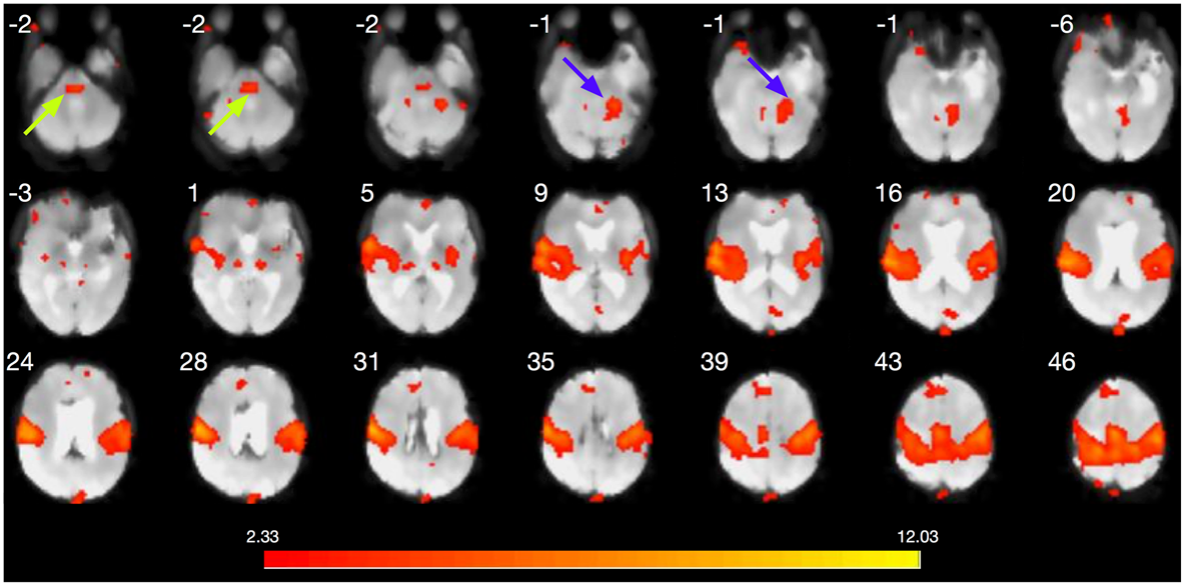
Sensorimotor resting state network identified using an ICA approach and the FSL MELODIC tool. Atypical involvement of upper pontine structures is indicated by green arrows; and of the left cerebellar hemisphere by blue arrows. (image in conventional radiological orientation: image left = patient right; the site of the previous tumour resection in the left temporal lobe is discernable). Images labelled with z coordinate in MNI space. Numbers on the threshold bar relate to t values

Results of deterministic tractography with SMA seeds are shown in Fig. 2a. A striking symmetrical connectivity between the SMAs via the pons is seen in the patient (Fig. 2a left). This pontine-level connectivity is also detectable in some controls (1, 3 and particularly 4) although the usual transcallosal connectivity remains dominant in these cases (Fig. 2a right). Figure 3 shows functional correlates of this structural connectivity as reflected in the patient’s sensorimotor resting state network: pontine and left cerebellar hemisphere nodes are evident in the patient which are not present in this network in the literature [10].

Results of tractography studies with cerebellar hemisphere seeds are shown in Fig. 2b. In this figure, crossed connections are shown in yellow (left cerebellum to right SMA) and blue (right cerebellum to left SMA). The patient shows a striking right cerebellar hemisphere to left SMA connectivity crossing the midline at upper pontine level (Fig. 2b left) not seen in controls (Fig. 2b right).

## Conclusions

The anarchic hand phenomenon is poorly understood. Our data would support the hypothesis that callosal injury hinders control of the non-dominant hand by the dominant hemisphere [11]. The observation that the non-dominant hand is typically the anarchic hand may be because non-dominant hand movements are normally associated with a more bilateral activation of motor and premotor areas [12]. Anarchic hand syndrome has been reported in the dominant hand in situations of overt damage to the contralateral medial frontal cortex [13]. According to the dual premotor system theory, the medial premotor system (supplementary motor area and cingulate cortex) is responsible for internally generated (“willed”) movements, whereas the lateral premotor system generates movements in response to environmental cues. Damage to the medial premotor system leaves the contralateral hand vulnerable to external cues rather than internally generated control, resulting in an anarchic hand [14]. Although overt medial frontal damage was not evident in our case, altered structural and functional connectivity was demonstrable between superior frontal and cingulate structures respectively.

We are unaware of any reports in children younger than nine. To our knowledge anarchic hand has never been reported as a feature of congenital absence of the corpus callosum. Soman et al. [15] report a case of delayed emergence of anarchic hand symptoms eight years after a right middle cerebral artery territory stroke acquired at age three years, implying a necessary degree of callosal maturation of function before the phenomenon can occur.

It remains unclear why anarchic hand phenomena are intermittent. Although presumed epileptic paroxysmal alien hand phenomena have been reported [16], situational cues may be more pertinent. If the right hemisphere is “instructing” the non-dominant hand based on environmental cues, the outcome will only sometimes be in conflict with the left hemisphere.

As in our case, anarchic hand phenomena are commonly reported to improve over time, even after total commissurotomy [17]. The mechanisms of this resolution are unclear. Uddin et al. [18] proposed compensatory coordination by subcortical mechanisms. Our case shows striking connectivity from SMA seeds to pontine structures bilaterally (Fig. 2a). We suggest these are enhanced efferent projections as the first part of a feed-forward cerebrocerebellar system [19], with connectivity between SMAs re-established via a trans-cerebellar route (Fig. 2b) (rather than directly at pontine level). This would be consistent with the demonstration of atypical pontine and cerebellar involvement in the DMN (Fig. 3). It has been proposed that SMA injury can give rise to anarchic hand phenomena by loss of an inhibitory effect on primary motor cortex [20]: our findings would be consistent with this model if one hypotheses partial restoration of an inhibitory role of the right hemisphere SMA in controlling the left hand via a feed-forward pathway originating in the left SMA via the pathway identified in Fig. 2b and the right cerebellar hemisphere. It has been shown that plasticity can arise from changes in white matter microstructure as well as more conventional mechanisms of synaptic plasticity [21].

An important limitation of this study is that the studies were performed at a single time, when symptoms had largely resolved. The demonstrated findings could underlie either the occurrence of the anarchic hand phenomenon or its resolution. We hypothesise that the reduced connectivity between superior frontal areas and cingulate gyri contributed to the onset of the anarchic hand symptoms. The enhanced indirect connections between the SMAs may represent an adaptive response contributing to symptom resolution.

## Consent

Written informed consent was obtained from the patient for publication of this Case report and any accompanying images. Although fifteen at the time of the studies she was over sixteen at the time of the decision to submit this case report and gave consent in her own right. A copy of the written consent is available for review by the Editor of this journal.

## Abbreviations

DTI: Diffusion tensor imaging
EPI: Echo-planar imaging
FDR: False discovery rate
SMA: Supplementary motor area
TBI: Traumatic brain injury
